# The effects of proteins released from silk mat layers on macrophages

**DOI:** 10.1186/s40902-018-0149-1

**Published:** 2018-05-25

**Authors:** Ju-Won Kim, You-Young Jo, Hae Yong Kweon, Dae-Won Kim, Seong-Gon Kim

**Affiliations:** 1 Department of Oral and Maxillofacial Surgery, Sacred Heart Hospital, Hallym University, Jukheon gil 7, Gangneung, Gangwondo 25457 Republic of Korea; 2 Sericultural and Apicultural Materials Division, National Academy of Agricultural Science, Wanju-gun, 55365 Republic of Korea; 3 Department of Oral Biochemistry, College of Dentistry, Gangneung-Wonju National University, Gangneung, 25457 Republic of Korea; 4 Department of Oral and Maxillofacial Surgery, College of Dentistry, Gangneung-Wonju National University, Gangneung, 25457 Republic of Korea

**Keywords:** Silk, Inflammation, Angiogenesis inducing agents

## Abstract

**Background:**

The objective of this study was to evaluate the changes in gene expression after incubation of cells with proteins released from different silk mat layers.

**Methods:**

A silk cocoon from *Bombyx mori* was separated into four layers of equal thickness. The layers were numbered from 1 to 4 (from the inner to the outer layer). The proteins were released by sonication of a silk mat layer in normal saline. The concentration of proteins was determined by spectrophotometry. They were incubated with RAW264.7 cells, and changes in the expression of genes were evaluated by cDNA microarray analysis and quantitative reverse transcriptase-polymerase chain reaction (qRT-PCR).

**Results:**

Layer 1 and 4 groups had higher protein concentrations compared to those in layer 2 and 3 groups. The genes associated with inflammation and angiogenesis showed significantly higher expression in layer 1 and 4 groups. The results of qRT-PCR were in agreement with those of the cDNA microarray analysis.

**Conclusions:**

The silk mat from the middle portion of the silkworm cocoon yielded a lower protein release and caused an insignificant change in the expression of genes that are associated with inflammation and angiogenesis.

## Background

A silk mat is produced from a *Bombyx mori* silkworm cocoon by separating layers [1, 2]. Because the silkworm cocoon is mainly composed of proteins fibroin and sericin, the silk mat has composition similar to that of the silkworm cocoon [3]. The concentration of fibroin is not much different among the layers [4]. Nonetheless, sericin content generally increases from the inner layer to the outer layer [5]. Aside from these two proteins, seroin and protease inhibitors can be found in the silk mat [4]. The concentrations of these proteins are also different among layers. Seroin and several protease inhibitors are enriched in the innermost layer [4]. When silk mat is implanted into the body, the biological response to silk mat may be different to its origin. However, there has been few studies to illuminate these differences.

Biological response to silk mat will be determined by released protein from silk mat surface or proteins undergone proteolysis by macrophages. Sericin is an adhesive protein and encompasses fibers [6, 7]. Sericin is water soluble and is released from a silk mat in a fragmented form [8]. Because the concentrations of sericin are different among the layers, the released protein concentration is also different among the layers [9]. Seroin and some protease inhibitors are hydrophilic [4] and can be released from each layer.

The amount of protein released into normal saline is lower for the middle portion of the silkworm cocoon [3, 9]. These protein solutions are mainly composed of sericin, which is known to be a degumming product [9]. Nevertheless, that study did analyze the proteins between 15 and 50 kDa [9]. For example, the molecular weight of seroin is under 15 kDa [4]. Therefore, proteins other than sericin may be present in the solution containing released proteins. These protein solutions increase the expression level of tumor necrosis factor-α (TNF-α) in macrophages [9]. The expression level of TNF-α is generally proportional to the protein concentration in the solution being applied [9]. The detailed TNF-α expression pattern is different between the solutions derived from the innermost layer and from the outermost layer [9]. Because the concentrations of seroin and of protease inhibitors are also different among the layers [4], the difference in expression patterns may be due to the compositional dissimilarity.

Aside from TNF-α quantification, there have been few studies about the gene expression changes in macrophages under the influence of the silk mat-derived soluble proteins. Recently, a silk mat was approved by the Korean Food and Drug Administration for clinical trials (SPENSER-TS101 approved at November 27, 2015). As a silk mat is at the final stage of evaluation for clinical application, detailed information on the released proteins’ effects are important. cDNA microarrays have been widely used for the screening of overall genetic expression changes in cells or tissues [10]. The purpose of this study was to screen gene expression changes after application of a silk mat-derived protein solution to macrophages. Quantitative reverse transcriptase-polymerase chain reaction (qRT-PCR) analysis was performed on selected genes to confirm the microarray results.

## Methods

### Preparation of a solution of proteins released from a silk mat

Cocoons of *Bombyx mori* were kindly gifted by the Rural Development Administration (Wanju, Korea). A cocoon was cut and separated into four layers of the same thickness. Each layer was designated as layer 1 through layer 4 (from the inner to the outer layer). To acquire the proteins released from each silk layer, the silk mats of each layer (2 g) were placed into 50 mL of normal saline at 25 °C. To accelerate protein release from each silk mat, normal saline was sonicated with a silk mat for 4 h. After removal of the silk mat, the protein concentration in normal saline was determined by spectrophotometry at 280 nm. The standard curve was constructed via measurement of bovine albumin concentration.

### Cell cultures and application of a protein solution

RAW264.7 cells (KCLB No. 40071) were used for studying gene expression profiles under the influence of the proteins released from a silk mat. The detailed procedure was in accordance with our previous publication [9]. The culture medium was Dulbecco’s modified Eagle’s medium-high glucose (PAA Laboratories, Linz, Austria) supplemented with 1% of a penicillin/streptomycin solution (100×) and 10% of fetal bovine serum (FBS). The cells were grown to 80% confluence at 37 °C in an atmosphere containing 5% of CO_2_ and at 99% relative humidity. Then, the culture medium was changed to the same composition without FBS. After measurement of the protein concentrations, the same volume of a protein solution from each layer (750 μL) was added into the culture medium of RAW264.7 cells. The same amount of normal saline was added to the medium as a control. During incubation with the protein solution, total RNA from each group was isolated at 2 and 8 h after initiation of the incubation. For qRT-PCR, total RNA from each group was collected at 2, 8, and 24 h during the incubation.

### cDNA microarray analysis and qRT-PCR

The quality of total RNA was evaluated on an Agilent 2100 Bioanalyzer (Agilent Technologies, Santa Clara, CA, USA). RNA integrity number of all the samples was greater than 7.0 and met the criterion for the subsequent microarray analysis. Commercially available microarray chips for mouse genes (Agilent MouseGE 4 × 44 K, Agilent Technologies) were used. By means of Agilent’s Low RNA Input Linear Amplification kit plus (Agilent Technologies), extracted total RNA was subjected to amplification and labeling. After unhybridized probes were washed out, chips were scanned, and the scanned images were analyzed in the Feature Extraction Software (Agilent Technologies). Normalization and clustering were performed in GeneSpring software (Agilent Technologies). For comparative purposes, gene expression in the untreated control was also analyzed. The comparison of the expression of genes between each layer group and the no-treatment control was performed in the generated scatter plot.

Three genes among inflammation-associated genes were selected and analyzed by qRT-PCR for confirmation. The primers for the interleukin 1β (*Il1β*) were F: 5′-AACCATGGCACATTCTGTTC-3′ and R: 5′-AGGTAAGTGGTTGCCCATCA-3′. Those for interleukin 18 (*Il18*) were F: 5′-GTTCTCTGTGGTTCCATGCT-3′ and R: 5′-CTGGAGGTTGCAGAAGATGT-3′. That for *Tnfα* were F: 5′-ACAAGATGCTGGGACAGTGA-3′ and R: 5′-GCTCCAGTGAATTCGGAAAG-3′. That for glyceraldehyde 3-phosphate dehydrogenase (*Gapdh*) were F: 5′-GGCATTGCTCTCAATGACAA-3′ and R: 5′-ATGTAGGCCATCAGGTCCAC-3′. Reverse-transcribed total RNA served as a template in each PCR. The threshold cycle (Ct) values were determined. Relative expression was calculated as a ratio of the selected gene’s expression to the expression of *Gapdh*. Measurements were taken three times, and the average value served for comparison.

### Statistical analysis

Cluster analysis was carried out in Gene Cluster 3.0 (Stanford University, CA, USA). Genes were filtered, and data were normalized. For hierarchical analysis, genes were clustered, and uncentered correlation served as a similarity metric. The clustering method was centroid linkage. For the comparison of gene expression levels by qRT-PCR, ANOVA was performed. As the post hoc analysis, Bonferroni’s test was conducted. The significance level was set to *P* < 0.05.

## Results

**Fig. 1:**
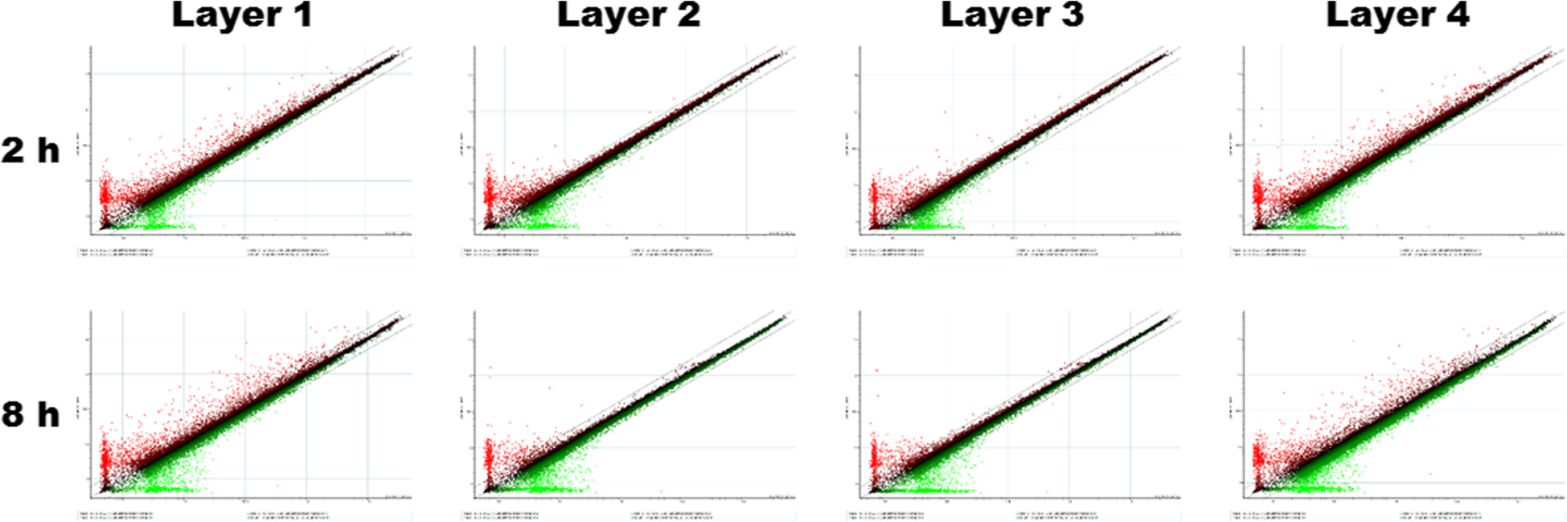
A sigma plot for each group. The gene expression ratio between saline control and each silk mat layer is shown as a single spot. Most gene data were collected on the line having inclination of 1.0. The number of genes that underwent more than 2-fold changes in expression was much greater in layer 1 and 4 groups than in layer 2 and 3 groups

**Fig. 2:**
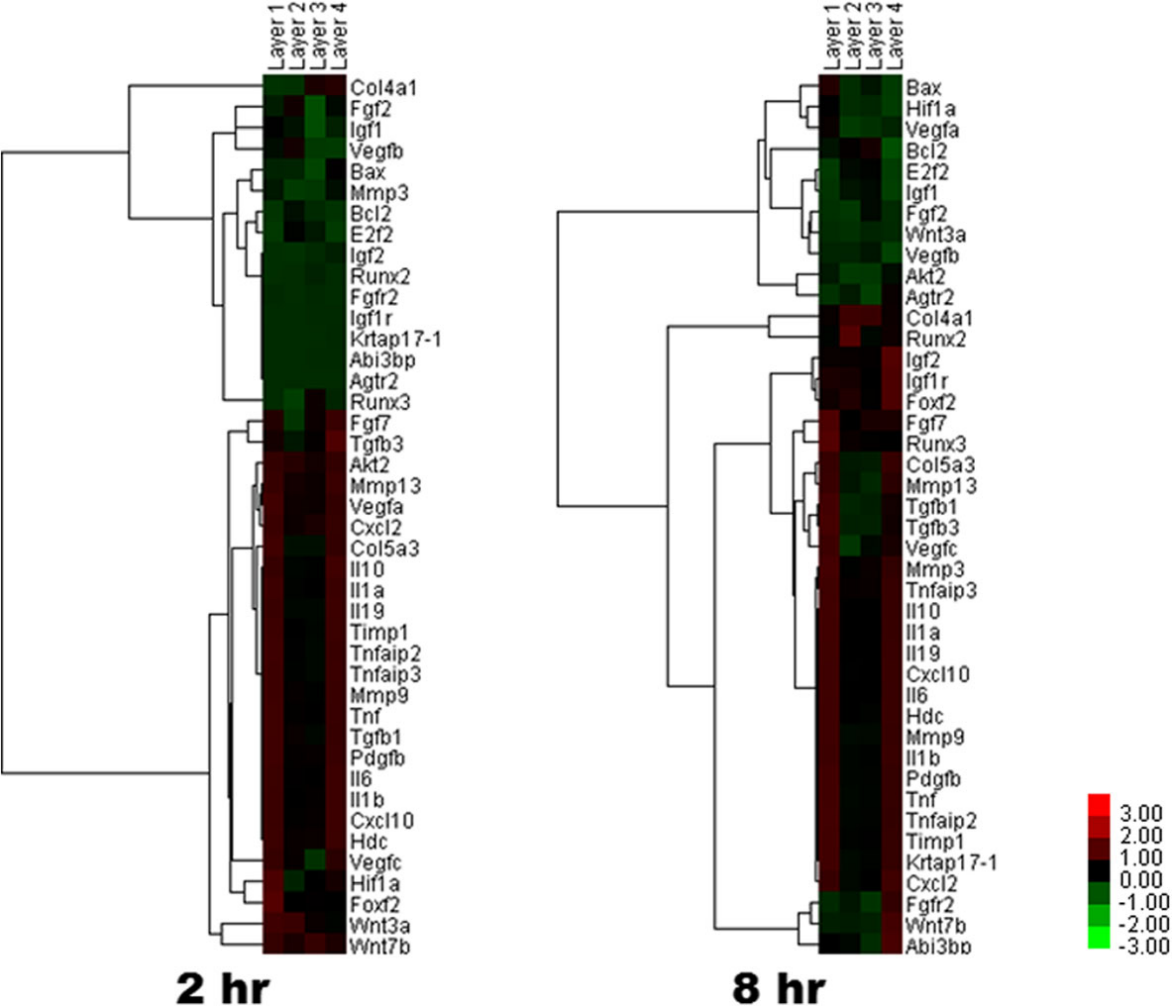
A gene tree with cluster analysis. The genes associated with inflammation and angiogenesis were highly expressed in layer 1 and 4 groups at 2 h. The elevated expression of some genes persisted until 8 h

The protein concentration in each solution was 10.08, 3.37, 3.47, and 8.41 μg/mL for layer groups 1, 2, 3, and 4, respectively. After addition of each solution to cell culture, changes of gene expression were studied. The scatter plots for each array are shown in Fig. 1. The number of genes manifesting significant fold changes (/fold ratio/> 2) was greater in layer 1 and 4 groups than in layer 2 and 3 groups. Selected genes were subjected to cluster analysis (Fig. 2). The genes in the *transforming growth factor-β* (*TGF-β*) family and the genes associated with inflammation showed higher levels of expression in layer 1 and 4 groups than in layer 2 and 3 groups. Most genes showed a stable level of expression until 8 h. Nevertheless, the expression of some genes decreased at 8 h.

Table 1 shows the summary of significantly differentially expressed genes. Layer 1, 2, and 4 groups showed significantly lower expression of *fibroblast growth factor-2* (*Fgf2*) at 8 h after addition of a protein solution (*P* < 0.05). The layer 1 group showed significantly higher mRNA expression of *Fgf7* at 8 h (*P* < 0.05). In contrast, the expression of insulin-like growth factor-1-receptor (*Igf1r*) was significantly higher in all groups at 2 h (*P* < 0.05). The gene expression levels of *interleukin* and *Tnf* families were higher in layer 1 and 4 groups. *Matrix metalloproteinase-3* (*Mmp3*) and *Mmp9* also manifested significantly higher gene expression in layer 1 and 4 groups at 8 h (*P* < 0.05). *Platelet-derived growth factor* (*Pdgf*) is associated with angiogenesis and showed significantly higher gene expression in layer 1 and 4 groups at 2 and 8 h (*P* < 0.05). *Hypoxia-inducible factor-1-α* (*Hif1α*) was significantly upregulated in the layer 1 group at 2 h (*P* < 0.05). The Wnt signaling pathway is closely associated with angiogenesis. *Wnt3a* was overexpressed in layer 1 and 2 groups at 2 h but was significantly underexpressed at 8 h in all groups (*P* < 0.05). In the case of *Wnt7b*, it was significantly overexpressed in all groups at 2 h, but the elevated level of expression persisted only in the layer 4 group (*P* < 0.05).

**Table 1:** Summary of microarray results

Gene	Observation	Layer 1	Layer 2	Layer 3	Layer 4
*Fgf2*	2 h	− 0.731	0.450	−1.961*	− 0.247
	8 h	− 2.731*	− 3.140*	− 0.591	− 2.441*
*Fgf7*	2 h	0.483	− 0.720	0.180	0.802
	8 h	1.772	− 0.001	0.575	0.640
*Igf1r*	2 h	− 3.106*	− 3.101*	− 2.943*	− 2.953*
	8 h	0.087	0.067	− 0.002	0.246
*Col4a1*	2 h	− 0.087	− 0.083	0.048	0.066
	8 h	0.364	2.300*	2.145*	0.484
*Fgfr2*	2 h	− 3.857*	− 4.213*	− 3.848*	− 4.119*
	8 h	− 0.126	− 0.067	− 0.157	0.157
*Hif1a*	2 h	1.435*	− 0.641	0.023	0.419
	8 h	− 0.023	− 0.599	− 0.518	− 0.817
*Il1b*	2 h	3.001*	0.067	0.269	3.102*
	8 h	3.233*	− 0.308	− 0.107	2.993*
*Il6*	2 h	5.252*	0.250	0.162	5.356*
	8 h	5.676*	0.058	− 0.080	5.085*
*Il19*	2 h	1.236*	− 0.134	− 0.168	1.298*
	8 h	3.463*	− 0.054	0.106	3.106*
*Mmp3*	2 h	− 0.864	− 2.123*	− 2.006*	− 0.511
	8 h	2.714*	− 0.113	0.436	1.954*
*Mmp9*	2 h	1.630*	0.173	− 0.063	1.514*
	8 h	1.171*	− 0.195	− 0.162	1.021*
*Pdgfb*	2 h	2.385*	0.166	0.241	2.155*
	8 h	2.455*	− 0.218	− 0.137	2.021*
*Tnfa*	2 h	1.462*	0.086	0.026	1.301*
	8 h	1.443*	− 0.170	− 0.110	1.182*
*Tnfaip3*	2 h	2.231*	− 0.084	− 0.254	2.119*
	8 h	3.420*	0.378	0.446	2.957*
*Wnt3a*	2 h	4.117*	3.730*	0.666	− 0.270
	8 h	− 3.989*	− 4.493*	− 3.257*	− 3.897*
*Wnt7b*	2 h	3.949*	2.108*	4.070*	2.147*
	8 h	− 0.630	− 0.570	− 0.704	1.458*

*P < 0.05 when compared to the saline-treated control

*Fgf* fibroblast growth factor, *Col* collagen, *Igf* insulin-like growth factor, *Hif* hypoxia-inducible factor, *Il* interleukin, *Mmp* matrix metalloproteinase, *Pdgf* platelet-derived growth factor, *Tnf* tumor necrosis factor

**Fig. 3:**
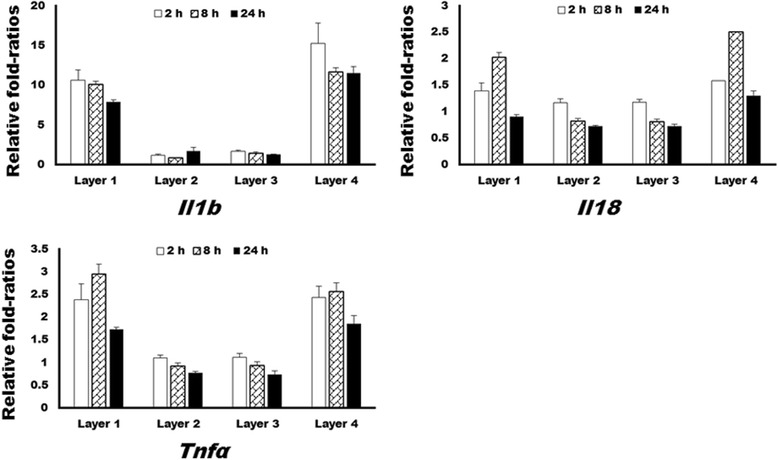
The results of quantitative reverse-transcriptase polymerase chain reaction (qRT-PCR) analysis of selected genes. The expression of *Il1β*, *Il18*, and *Tnfα* at 2 and 8 h was significantly higher in layer 1 and 4 groups than in layer 2 and 3 groups (*P* < 0.05). The expression of *Il1β* and *Tnfα* at 24 h was significantly higher in layer 1 and 4 groups than in layer 2 and 3 groups (*P* < 0.05)

For confirmation of the microarray results, qRT-PCR was conducted, and the results are demonstrated in Fig. 3. The expression of *Il1b*, *Il18*, and *Tnfα* was significantly higher in layer 1 and 4 groups at 2, 8, and 24 h after addition of the protein solution (*P* < 0.05). Compared to layer 1 and 4 groups, those expression levels in layer 2 and 3 groups manifested insignificant changes throughout the observation period.

## Discussion

In this study, a gene expression profile of macrophages was evaluated by cDNA microarray and qRT-PCR analyses. The proteins released from layers 1 and 4 increased the expression levels of genes associated with angiogenesis and inflammation. The main protein in the solution was likely sericin because 20–30% of a silk mat is composed of sericin, and proteins other than sericin and fibroin constitute less than 1–2% of a silk mat [5]. Therefore, these differences among groups may be due to the differences in sericin content among the layers.

### Genes associated with inflammation

Kundu et al. [11] reported that a sericin-coated dental implant did not significantly increase TNF-α and IL-1β expression in macrophages. Low level of sericin does not significantly increase TNF-α, but high level of sericin increases TNF-α significantly [9]. Accordingly, the expression levels of pro-inflammatory cytokines may be dependent on the concentration of applied sericin [9]. In case of *Il19*, its expression level in layer 1 and 4 groups was elevated at 8 h as compared to that at 2 h (Table 1). When the *Il19* gene is inactivated in mice, the expression level of pro-inflammatory cytokines increases [12].

The expression of *Hif1α* is elevated by pro-inflammatory cytokines such as IL1Α, IL6, and TNFα [13]. The expression of *Hif1α* was elevated here at 2 h after addition of the protein solution in the layer 1 group. If a HIF1Α inhibitor is applied, then protein expression of IL6 and TNFα decreases [14]. Accordingly, there is a close association between *Hif1α* expression and pro-inflammatory cytokines’ expression [15].

### Genes associated with angiogenesis

For vascular regeneration, the basement membrane of endothelial cells should undergo proteolysis, and MMPs are involved in this process [16]. The degradation of the basement membrane proteins can cause a release of free vascular endothelial growth factor (VEGF) [17]. In the present study, *Mmp3* and *Mmp9* showed elevated levels of gene expression in layer 1 and 4 groups (Table 1). *Wint3a* and *Wint7b* were significantly overexpressed at 2 h after addition of a protein solution in layer 1 and 4 groups (*P* < 0.05). The expression of *Wnt3a* is closely related to macrophage-mediated angiogenesis under pathological conditions [18]. Without WNT7B, transcription of *Vegfa* is strongly suppressed [19]. Consequently, the gene expression levels of *Mmp3*, *Mmp9*, and *Pdgfb* were high in layer 1 and 4 groups, which showed high levels of *Wint3a* and *Wint7b* expression. All of them are closely related to angiogenesis.

Hypoxic stress can increase the expression of *Pdgfb* in hepatic cancer cells, and this change leads to cellular proliferation and elevated *Vegf* expression [20]. When zoledronate is applied to pre-osteoclasts, the *Pdgfb* expression decreases and results in suppressed angiogenesis [21]. In osteogenesis, angiogenesis is a vital component, and PDGFB is the main regulator [22]. Besides, PDGFB is important for the healing process after vascular injury [23]. *Hif1α* is induced by hypoxic stress and associated with angiogenesis [13]. In our study, *Hif1α* and *Pdgfb* were overexpressed at 2 h in the layer 1 group (Table 1). This group’s increased levels of gene expression seemed to be due to the activation of angiogenesis induced by silk mat-derived proteins.

## Conclusions

Silk mat-derived protein solution increased the genes associated with inflammation and angiogenesis in macrophages as originated in a layer-dependent manner. For the development of biomaterials from a silk mat, different biological properties of each layer should be considered.

## Abbreviations

Fgf: Fibroblast growth factor
Gapdh: Glyceraldehyde 3-phosphate dehydrogenase
Hif1α: Hypoxia-inducible factor-1-α
Igf1r: Insulin-like growth factor-1-receptor
Il: Interleukin
Mmp: Matrix metalloproteinase
Pdgf: Platelet-derived growth factor
qRT-PCR: Quantitative reverse transcriptase-polymerase chain reaction
TGF-β: Transforming growth factor-β
TNF-α: Tumor necrosis factor-α
VEGF: Vascular endothelial growth factor
